# Repeated doses of Praziquantel in Schistosomiasis Treatment (RePST) – single versus multiple praziquantel treatments in school-aged children in Côte d’Ivoire: a study protocol for an open-label, randomised controlled trial

**DOI:** 10.1186/s12879-018-3554-2

**Published:** 2018-12-14

**Authors:** P. T. Hoekstra, M. Casacuberta Partal, A. S. Amoah, L. van Lieshout, P. L. A. M. Corstjens, S. Tsonaka, R. K. Assaré, K. D. Silué, A. Meité, E. K. N’Goran, Y. K. N’Gbesso, M. Roestenberg, S. Knopp, J. Utzinger, J. T. Coulibaly, G. J. van Dam

**Affiliations:** 1 Department of Parasitology, Leiden University Medical Center, Leiden, the Netherlands; 2 Department of Cell and Chemical Biology, Leiden University Medical Center, Leiden, the Netherlands; 3 Department of Medical Statistics and Bio-Informatics, Leiden University Medical Center, Leiden, the Netherlands; 4 Swiss Tropical and Public Health Institute, Basel, Switzerland; 5 University of Basel, Basel, Switzerland; 6 Unité de Formation et de Recherche Biosciences Université Félix Houphouët-Boigny, Abidjan, Côte d’Ivoire; 7 Centre Suisse de Recherches Scientifiques en Côte d’Ivoire, Abidjan, Côte d’Ivoire; 8 Programme National de Lutte contre les Maladies Tropicales Négligées à Chimiothérapie Préventive, Abidjan, Côte d’Ivoire; 9 Departement d’Agboville, Centre de Santé Urbain d’Azaguié, Azaguié, Côte d’Ivoire

**Keywords:** Schistosomiasis, *Schistosoma mansoni*, Praziquantel, Treatment efficacy, Diagnostic test, Kato-Katz, CAA, CCA, PCR

## Abstract

**Background:**

Large scale administration of the anthelminthic drug praziquantel (PZQ) to at-risk populations is the cornerstone of schistosomiasis control, although persisting high prevalence of infections in some areas and growing concerns of PZQ resistance have revealed the limitations of this strategy. Most studies assessing PZQ efficacy have used relatively insensitive parasitological diagnostics, such as the Kato-Katz (KK) and urine-filtration methods, thereby overestimating cure rates (CRs). This study aims to determine the efficacy of repeated PZQ treatments against *Schistosoma mansoni* infection in school-aged children in Côte d’Ivoire using the traditional KK technique, as well as more sensitive antigen- and DNA-detection methods.

**Methods:**

An open-label, randomised controlled trial will be conducted in school-aged children (5 to 18 years) from the region of Taabo, Côte d’Ivoire, an area endemic for *S. mansoni*. This 8-week trial includes four two-weekly standard doses of PZQ in the “intense treatment” intervention group and one standard dose of PZQ in the “standard treatment” control group. The efficacy of PZQ will be evaluated in stool samples using the KK technique and real-time PCR as well as in urine using the point-of-care circulating cathodic antigen test and the up-converting phosphor, lateral flow, circulating anodic antigen assay. The primary outcome of the study will be the difference in CR of intense versus standard treatment with PZQ on individuals with a confirmed *S. mansoni* infection measured by KK. Secondary outcomes include the difference in CR and intensity reduction rate between the intense and standard treatment groups as measured by the other diagnostic tests, as well as the accuracy of the different diagnostic tests, and the safety of PZQ.

**Discussion:**

This study will provide data on the efficacy of repeated PZQ treatment on the clearance of *S. mansoni* as measured by several diagnostic techniques. These findings will inform future mass drug administration policy and shed light on position of novel diagnostic tools to evaluate schistosomiasis control strategies.

**Trial registration:**

The study is registered at EudraCT (2016–003017-10, date of registration: 22 July 2016) and (NCT02868385, date of registration: 16 August 2016).

**Electronic supplementary material:**

The online version of this article (10.1186/s12879-018-3554-2) contains supplementary material, which is available to authorized users.

## Background

Schistosomiasis is still a major public health problem in many tropical countries, particularly in Africa where more than 90% of the global burden of schistosomiasis occurs [1]. Large-scale administration of the anthelminthic drug praziquantel (PZQ) to at-risk populations has become the cornerstone of schistosomiasis control [2, 3]. This strategy – known as preventive chemotherapy – has been successful in reducing infection intensities, and hence morbidity [4, 5]. As morbidity is a result of cumulative exposure to a high number of schistosomes, school-aged children bear the largest burden of disease because they carry the highest intensity infections [6]. Therefore, mass drug administration (MDA) of preventive chemotherapy targets school-aged children primarily [6, 7]. These children are intermittently treated with a single oral dose of 40 mg/kg PZQ, the frequency of which depends on the prevalence of the infection in the community [8]. Target populations are divided into high-, moderate- and low-risk communities in which school-aged children are treated with PZQ once a year, once every 2 years or twice during their primary schooling age, respectively [9]. PZQ is the drug of choice for the treatment of all forms of schistosomiasis due to its high efficacy and excellent safety profile [10].

Observed cure rates (CRs) after a single dose of PZQ treatment (40 mg/kg) range between 42 and 79% for *Schistosoma mansoni* and between 37 and 93% for *S.haematobium* in school-aged children [11]. A second dose of PZQ at a later time point can increase the CR up to 93% for *S.mansoni* [11–13] and up to 99% for *S. haematobium* [11]. However, the estimated efficacy of PZQ is highly dependent on the diagnostic tool used to measure CRs. Most studies have used traditional parasitological methods, such as the Kato-Katz (KK) and urine-filtration (UF) methods based on microscopy and determining the presence/excretion of eggs. These methods lack sensitivity for diagnosing low level infections and as such overestimate CRs [14, 15]. More sensitive diagnostic tools for schistosomiasis which are currently available and can be implemented in the field, are much more suited to evaluate the efficacy of PZQ and alternative dose regimens. For example, the commercially available point-of-care circulating cathodic antigen (POC-CCA) test, which indicates active worm infection by detection of parasite CCA in urine, has shown a high diagnostic accuracy for *S.mansoni* with a sensitivity ranging between 78 and 92% and specificity approaching 100% [15–17]. Over the past 10 years, this test has been widely evaluated in sub-Saharan Africa and is now recommended as the first line diagnostic to map schistosome prevalence and facilitate preventive chemotherapy strategic decision-making [7, 16, 18]. In addition, there is a pressing need for ultra-sensitive diagnostic tools for areas where prevalence and infection intensity have been reduced to very low levels. Such diagnostic tools are needed to confirm interruption of transmission and possibly elimination of schistosomiasis. The circulating anodic antigen (CAA) detection assay fulfills these requirements. This assay measures parasite antigen both in urine and serum using an ultrasensitive reporter technology (up-converting phosphor particles, UCP) in combination with common immunochromatography, lateral flow (LF). This UCP-LF CAA assay has shown high sensitivity and specificity for all four main schistosome species (*S.haematobium*, *S.japonicum*, *S.mansoni* and *S.mekongi*) [19–22]. In addition to the UCP-LF CAA assay, highly specific and sensitive molecular polymerase chain reaction (PCR) techniques that detect parasite-specific DNA in stool and urine have also become available [16]. The combination of worm-derived antigens and egg-derived nucleic acids, are envisaged to further increase the sensitivity and specificity of the diagnostic toolbox and allow for a comprehensive assessment of PZQ efficacy, with respect to both parasite worm dynamics and fecundity.

### Rationale

There is a need for a re-evaluation of previously established PZQ CRs to provide evidence for continuing mass distribution of PZQ in high risk communities. Previously, CRs may have been overestimated due to insensitive diagnostic tools, whereas continuing reinfections and the fact that PZQ has little activity on immature worms, might have led to an underestimation of the therapeutic effect [23–26]. Repeated treatment with PZQ at short intervals (e.g. 2–8 weeks) in areas with ongoing transmission will more effectively target non-susceptible schistosomula as they will have matured into drug susceptible worms during this period [11, 27], thereby increasing the drug effectiveness. As the short metabolic half-life of PZQ may also limit its effectiveness by suboptimal plasma levels, repeated dosing will increase the chance that all worms are affected [28]. Whether this will be reflected in a significant decrease in schistosome prevalence and the potential to interrupt transmission, remains to be investigated [11, 15, 29]. In this study, we will evaluate the effect of multiple doses of PZQ on parasite clearance and tolerance in individuals infected with *S.mansoni*.

The primary objective of this study is to determine the efficacy of PZQ treatment for clearing *S.mansoni* infections in a multiple dose regimen (standard dose, four times, two-week intervals) using the KK technique. Secondary objectives include determining the efficacy of PZQ for clearing *S.mansoni* infections in a multiple dose regimen using DNA- and antigen-detection techniques, evaluation the safety of PZQ and determining the accuracy of the different diagnostic tests used in this study. Exploratory objectives include modelling the effect of multiple PZQ treatments on the transmission of schistosomiasis as well as modelling the biological effects of multiple PZQ treatments on individual worm burden, egg load and re-infection rates.

## Methods

### Study design

**Fig. 1:**
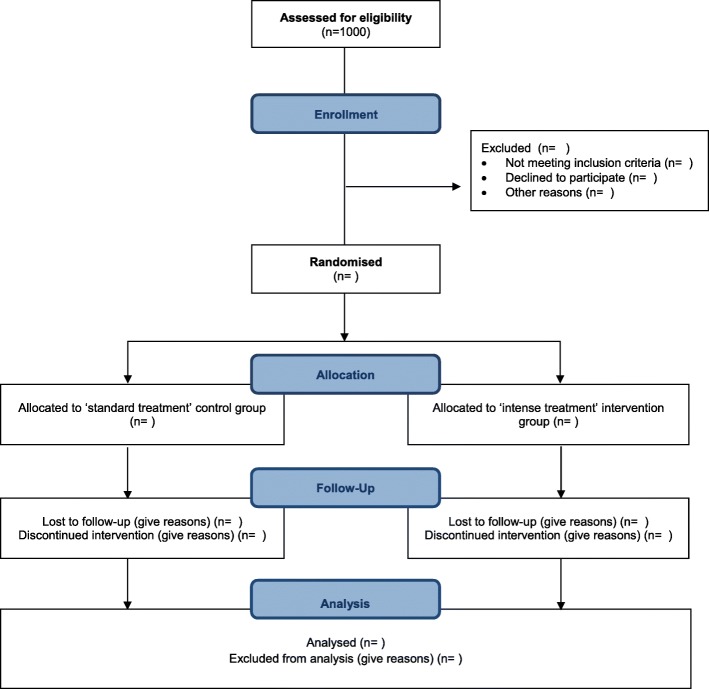
Flow-diagram

To evaluate the repeated doses of PZQ in schistosomiasis treatment (RePST), an open-label, randomised controlled trial will be conducted, with the primary aim to compare the efficacy of one versus four doses of PZQ in *S. mansoni* infected school-aged children in Côte d’Ivoire, using the traditional KK thick smear technique as well as with more sensitive antigen- and DNA-detection methods (Fig. 1). After screening for eligibility, participants are randomised into two groups in a 1:1 ratio. Individuals assigned to the standard treatment group will receive a single dose of PZQ (40 mg/kg) at baseline (week 0) and will receive no further treatment until the final visit. Individuals assigned to the intense treatment group will receive four doses of PZQ at baseline (week 0) and at three other time points with 2-week intervals. Follow-up and sample collection will take place every week for a period of eight weeks. At the end of the study, all children from selected communities (including study participants) will be offered a standard dose of PZQ (single oral dose of 40 mg/kg) as well as albendazole (single oral dose of 400 mg) according to international guidelines of the World Health Organization (WHO), in coordination with the National Control Programme of Côte d’Ivoire (Programme National de Lutte contre les Maladies Tropicales Négligées à Chimiothérapie Préventive, PNLMTN-CP), for the treatment of schistosomiasis and soil-transmitted helminth infections, respectively.

### Study area and population

The study population will consist of school-aged children (5 to 18 years) from selected villages of Singrobo, Ahouaty and N’Denou in the Taabo health district, south-central Côte d’Ivoire. This district is part of the Taabo health and demographic surveillance system (HDSS) and is located approximately 150 km north of Abidjan and has been described in more detail previously [30, 31]. It comprises a small town, 13 villages and over 100 hamlets, with a total population of 42.480 inhabitants in 2013. The Taabo HDSS focuses, among other things, on neglected tropical diseases such as schistosomiasis and soil-transmitted helminths. Repeated cross-sectional epidemiological surveys on approximately 5–7% of the population and specific, layered-on haematological, parasitological and questionnaire surveys have been regularly conducted every year since 2009 within the Taabo HDSS [30].

#### Inclusion criteria

In order to be eligible to participate in this study, an individual must meet the following criteria:

- provide oral and signed assent as well as written informed consent signed by parents/legal guardian(s)
- have a confirmed *S. mansoni* infection (i.e. positive test result for POC-CCA and at least one positive KK thick smear);
- be between 5 and 18 years of age;
- have a good medical condition, as determined by the study physician based on biochemical, physical and clinical indicators (i.e. absence of acute or severe chronic disease);
- have received no PZQ treatment in the past 3 months; and
- be able and willing to provide multiple blood, stool and urine samples during the study.

#### Exclusion criteria

A potential participant who does not meet the inclusion criteria or who meets any of the following criteria will be excluded from participation in this study:

- have a confirmed *S. haematobium* infection (as determined by UF);
- have a known allergy to study medication (i.e. PZQ and albendazole); and
- is pregnant (confirmed by pregnancy HCG test) or lactating.

### Procedures

#### Screening and informed consent

Individuals will be assessed for eligibility during a 2-week baseline screening. This will include public meetings of awareness and health education, to allow all members of the communities to be well informed on the project. During these meetings, particular emphasis will be placed on modes of transmission, associated pathologies and risk factors related to schistosomiasis in particular and intestinal worms in general. After explaining the purpose of the study in the local language, potential participants will be asked to participate. After obtaining oral and written assent from the children and signed informed consent from their parents/legal guardians, standard demographic and other characteristics (e.g. age and sex) will be collected. Participants will be asked to provide a urine sample which will be tested on site immediately for *Schistosoma* infection by the POC-CCA urine test as well as with UF. The initial screening by POC-CCA will increase the likelihood of finding participants with a positive KK. Those with a positive POC-CCA test result (scoring 1+ or higher) as well as a negative UF result, will be asked to provide one stool sample which will be examined for *S.mansoni* eggs by triplicate KK. If at least one out of three smears is positive, the participant will be asked to undergo a biological, clinical and physical examination to determine whether he/she is in good health. For the biological exam, a blood sample will be obtained and tested for haematological indicators, liver function and renal function parameters. A study physician will perform the physical and clinical examinations, which will consist of checking the participant’s physical condition as well as determining if the participant has any chronic disease(s). All participants who meet the inclusion criteria will be enrolled and randomised.

#### Follow-up, sampling procedure and storage of samples

**Fig. 2:**
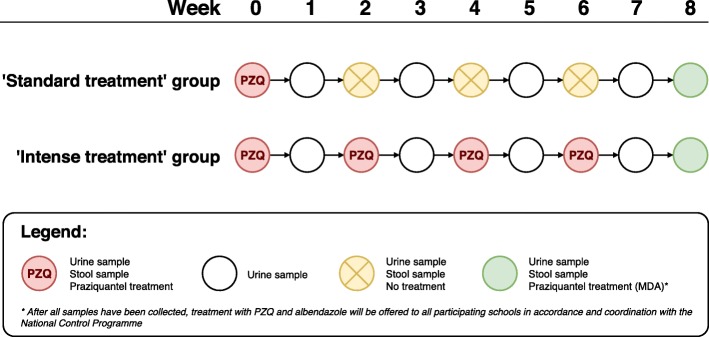
Schematic representation of follow-up, treatment and sampling procedures in the standard and intense treatment group.

After randomisation, follow-up will take place on the selected groups every week. Participants will be asked to provide one urine sample every week and one stool sample every two weeks. A more detailed and schematic representation is given in Fig. 2.

Each urine sample will be tested with the POC-CCA test and KK examination will be performed on each stool sample. An additional amount of sieved stool (300–400 μL of volume) will be mixed with 1 mL of 96% ethanol and stored for real-time PCR [32]. After every visit, all collected samples will be taken to the laboratory of the Centre Suisse de Recherches Scientifiques en Côte d'Ivoire (CSRS)-Fairmed in Taabo for temporary storage. Urine samples will be stored at − 20 °C and stool samples stored in ethanol. Once sample collection is completed, all samples will be transported from the CSRS-Fairmed laboratory in Taabo to CSRS in Abidjan. From all samples, one aliquot will be stored at CSRS in Abidjan for long-term storage and one aliquot will be sent to the Leiden University Medical Center (LUMC), the Netherlands, for additional testing with the UCP-LF CAA assay on urine samples and real-time PCR on stool samples.

#### Diagnostics

As part of the inclusion-exclusion process, several tests will be executed to determine eligibility for the study. Absence of pregnancy, if appropriate, will be confirmed with locally available pregnancy (HCG) tests. Infection with *S. haematobium*, will be determined by filtration of 10 mL of urine and microscopically examination, as described previously [33]. Finally, to verify a potential participant’s good health, a blood sample will be tested for basic biochemical and haematological parameters (e.g. urea, creatinine, liver enzymes and haemoglobin) in a qualified local laboratory.

All urine samples will be tested at the CSRS-Fairmed laboratory in Taabo using the POC-CCA assay (batch no.: 170522062, Rapid Medical Diagnostics, Pretoria, South Africa) as previously described [7, 17, 34]. At 20 min, valid tests will be scored as negative or positive according to a set of 10 novel artificial cassettes with inkjet-printed test lines representing negative and positive results depending on the intensity of the test line (artificial score 1–10). These novel artificial scores will be transformed into stratified positive scores of trace, 1+, 2+ or 3+. All tests will be read independently by two trained laboratory technicians. In case of discordant results, a third independent investigator will be consulted and results will be discussed until agreement is reached within 25 min. UF will be performed on urine samples collected at the final time point (week 8). Additionally, all urine samples will be examined at the LUMC using the UCP-LF CAA assay: a maximum of 2 mL urine will be analysed with a cut-off threshold of 0.1 pg/ml, as previously described [19, 35]. CAA results will be reported quantitatively in pg/ml.

From each stool sample triplicate KK thick smears will be prepared at the laboratory of CSRS-Fairmed in Taabo, using 41.7 mg templates, following standard protocols [36]. Briefly, three KK thick smears (A, B and C) will be prepared on microscope slides and examined quantitatively under a microscope by two independent, experienced laboratory technicians one (A), two (B) and three (C) days after preparation. Results will be reported as eggs per slide and converted to eggs per gram of faeces (EPG).

To detect the presence of *Schistosoma* DNA, real-time PCR analysis will be performed on the ethanol-preserved stool samples at the LUMC. Sample handling, DNA isolation and PCR analysis will be performed as described previously [37, 38]. The PCR output consists of a cycle-threshold (Ct) value, which represented the amplification cycle in which the level of fluorescent signal exceeded the background fluorescence, thereby indicating the amount of parasite-specific DNA in the sample that was tested.

#### Treatment and adverse events

Treatment of participants with PZQ will be administered by the study physician. The standard treatment group will receive a standard dose of PZQ (single oral dose of 40 mg/kg) at week 0, while the intense treatment group will receive PZQ at week 0, week 2, week 4 and week 6. PZQ treatment will be given after a light meal, as recommended by WHO [2], to minimize potential adverse events. The tablets will be administered under supervision of the study physician. If a participant vomits within 1.5 h following PZQ administration, another standard dose of PZQ will be given.

Participants will be monitored for adverse events at 3 h and 24 h after PZQ administration. An adverse event is defined as any undesirable experience occurring to a participant during the study, whether or not considered related to PZQ treatment. All adverse event intensities will be assessed by the study physician, following local guidelines and will be graded as mild, moderate or severe. Additionally, to monitor the functioning of the vital organs in the intense treatment group, participants will be asked to provide blood samples at weeks 3 and 7, after the second and fourth treatment, respectively, to be tested for haematological indicators, liver function and renal function parameters.

At the end of the study all school-aged children in the selected communities, including those who participated in the study and those who participated in the baseline screening but were not eligible to participate in the study based on inclusion/exclusion criteria as well as those who were not invited for baseline screening, will be offered PZQ and albendazole treatment according to and supplied by the PNLMTN-CP in Côte d’Ivoire.

#### Withdrawal

Participation is voluntary and participants can decide not to continue their participation in the study at any time for any reason if they wish to do so, without any consequences. The principal investigator can also decide to withdraw a participant from the study for any (urgent) medical reasons.

### Outcomes

The primary outcome for the study is:

- Difference in CR of one versus four standard doses of PZQ (given two weeks apart) in participants infected with *S.mansoni*, measured by KK at baseline compared to week 8.

Secondary outcomes are:

- Difference in CR of one versus four standard doses of PZQ in participants infected with *S.mansoni* as measured by the different diagnostic tests at baseline compared to week 8.
- Difference in CR of one versus two or three standard doses of PZQ as measured by the different diagnostic tests at baseline compared to weeks 4 or 6, respectively.
- Difference in intensity reduction rate (IRR) of one versus four standard doses of PZQ (given two weeks apart) on participants infected with *S. mansoni* measured by the different diagnostic tests at baseline compared to week 8.
- Difference in IRR between intervention and control group of one versus two or three standard doses of PZQ as measured by the different diagnostic tests at baseline compared to weeks 4 or 6, respectively.
- Sensitivity and specificity of the different diagnostic tests at different time points.
- Safety of repeated standard doses of PZQ.

### Outcome measures

The CR is defined as the proportion of participants who were *S. mansoni* egg positive at baseline and who became *S. mansoni* egg negative at week 8, as determined by KK. For the primary outcome, the CR in the standard treatment group will be compared to the CR in the intense treatment group. For the other diagnostic tests, the CR will be the proportion of participants who were positive by urine POC-CCA, urine UCP-LF CAA or stool PCR at baseline and who became negative at week 8. Differences between the standard and intense treatment group will be compared. The overall IRR in the intense and standard treatment groups will be calculated as the intensity of infection at week 8 compared to the intensity of infection at baseline, as determined by the different diagnostic tests (intensity referring to egg counts for KK, artificial score for POC-CCA, CAA level in pg/ml for UCP-LF CAA, Ct-value for real-time PCR). Additionally, the CR and IRR will be determined at intermediate time points after one versus two and one versus three standard doses of PZQ.

All adverse events occurring within 24 h after PZQ treatment will be recorded to evaluate the safety and tolerability of repeated PZQ treatment.

### Randomisation and blinding

All participants will be randomised at baseline in a 1:1 ratio, by an independent statistician. Local nurses and physicians will not be blinded to treatment. Study personnel, laboratory technicians and investigators will be blinded. Data analysis will be performed blinded to the intervention.

### Sample size calculation

The sample size estimation is based on the difference in CR of one versus four repeated standard doses of PZQ measured with KK 8 weeks after treatment. Based on previous, data we assume a CR of 66% after one standard dose of PZQ [11] and aim for an increased CR of 98.7% after four repeated doses of PZQ, leading to a sample size of 30 participants per group, with a power of 90% and a level of significance of 5% (2-tail) [39]. Because we anticipate a considerable loss to follow-up, we aim to include approximately 100 participants in each group, hence 200 participants in total.

Assuming a *S. mansoni* prevalence of approximately 25% by KK (unpublished data, Taabo HDSS survey February 2016) it is estimated that at least 1000 children will have to be screened to obtain a minimum of 200 KK positives in the selected region.

### Data management and statistical analysis

Each participant will be given a unique study identification number. Data will be collected using paper-based case report forms (CRFs) and will be double entered and managed by well-trained data entry personnel using the REDCap electronic data capture tools hosted at the Leiden University Medical Center, the Netherlands, via Emory University, Atlanta, GA, USA [40].

All analyses will be conducted blinded to the treatment allocations and will be performed using STATA version 12 (StataCorp; College Station, TX, USA) or IBM Statistical Package for Social Sciences version 23 (SPSS Inc., Chicago, Illinois, USA) or R language (R Foundation for Statistical Computing; Vienna, Austria). Response and dropout rates will be assessed and reported. Demographics and outcome parameters will be summarised using descriptive summary measures, expressed as mean (standard deviation) or median (interquartile range) for continuous variables depending on whether the data are normally distributed and frequencies (percentage) for categorical variables. The proportion of participants positive for intestinal schistosomiasis will be calculated as the proportion of participants who tested positive at various time points (e.g. baseline, week 1, week 2, etc.). These percentages positive for schistosomiasis will be reported separately for each diagnostic test. Baseline characteristics between the control and intervention groups will be compared using the X^2^ test for categorical variables and *t*-test or Mann-Whitney U for continuous variables depending on the distribution of the data.

For the primary and secondary outcomes, a repeated measurements analysis approach will be used [41]. With this approach, the correlation between the measurements collected from the same participant over time will be modelled using properly chosen correlation matrices. Based on this analysis the proportions cured at different time points will be estimated and differences between the intense and standard treatment group will be tested for statistical significance. Similarly, for the IRR the progression of the reduction in intensity will be estimated and differences between the two groups at different time points will be tested for statistical significance. This model-based approach is becoming a more popular method to properly analyse longitudinal data on treatment efficacy of PZQ among individuals as well as among groups of individuals [42–44]. Transformation of data will be applied if needed. The model will be adjusted for covariates such as age and sex. Although the assumption will be made that missing data will be missing at random, reasons for individuals missing treatment at each time-point will be recorded.

In the absence of a true ‘gold’ standard, the sensitivity and specificity for KK, POC-CCA, UCP-LF CAA and PCR will be estimated by using an imperfect ‘gold’ standard (based on assumptions of 100% specificity for KK, CAA and PCR, similarly to Knopp et al., 2015 [45]) as well as by using latent class analysis (LCA). LCA uses all available data to estimate the proportion of true positives that test positive for each test (i.e. the sensitivity of each test), the proportion of true negatives that test negative for each test (i.e. the specificity of each test), and the proportion of individuals truly positive in the study population (i.e. the infection prevalence within the study population), as described previously [46, 47]. The sensitivity, specificity, positive predictive value (PPV) and negative predictive value (NPV) of all diagnostic tests will be calculated with 95% confidence intervals (CIs). For all analysis, a *p* value of < 0.05 will be taken as the level for statistical significance.

### Data safety and monitoring board

An independent data safety and monitoring board (DSMB) has been established which will review safety data from the first and third treatment during the study and provide recommendations to the sponsor concerning the continuation of the study.

### Ethical considerations

The study is registered at ClinicalTrials.gov (reference number NCT02868385) as well as at the EU Clinical Trials Register (EudraCT, reference number 2016–003017-10) and will be conducted in accordance with the latest version of the Helsinki Declaration. The study has been approved by the National Ethics Committee of the Ministry of Health in Côte d’Ivoire (CNESVS, registration number 091–18/MSHP/CNESVS-km, 27 June 2018) as well as by the Direction de la Pharmacie, du Médicament et des Laboratoires de Côte d’Ivoire (DPML, registration number 99433-/MSHP/DGS/DPML/DAR and clinical trial number ECCI00618, 22 October 2018) and has been reviewed by the Ethics Committee of the Leiden University Medical Center in the Netherlands without any objections (CME, registration number P16.254, 11 January 2017). This study protocol, including the statistical analysis section, has been written before start of the trial. The data analysis of the main publication will follow this plan. The SPIRIT protocol checklist is given in Additional file 1.

## Discussion

The RePST trial is the first and currently the only clinical trial that will investigate the efficacy of four repeated standard doses of PZQ on the clearance of *S. mansoni* in a randomised trial design. Efficacy will be assessed by using several diagnostic techniques, including real-time PCR and the ultra-sensitive UCP-LF CAA assay. By using an extensive panel of diagnostics in a frequent post-treatment sampling schedule, this study is the first of its kind to comprehensively assess the efficacy of single as well as repeated doses of PZQ.

This study is a proof of concept study to determine the efficacy of repeated PZQ treatment in an endemic setting. The context is to provide evidence and tools for evaluating current schistosomiasis control approaches, as well as input for developing new strategies for individual cure and transmission interruption rather than population-wide morbidity control. Clearly this study is not aiming to design repeated PZQ treatment schedules for implementation in large-scale control programmes. Results will also prove to be highly relevant in individual test-and-treat approaches using non-invasive POC diagnostics, even in non-endemic settings. The in-depth analysis and validation of different diagnostic tools (before and) after treatment is essential to determine the effect of PZQ on different parasite-related parameters providing additional information on CRs, re-emergence of infections, and even on transmission potential. Accurate diagnosis of light intensity infections (after intensive chemotherapy) will also be crucial in the light of elimination of schistosomiasis now being a target in several endemic countries [48, 49] and clearly advocated by WHO [9].

## Trial status

This open-label, randomised controlled trial has started recruitment in October 2018. We envisage the sample collection period to be finished by the end of 2018, and sample processing and testing at the LUMC to start early 2019.

## Additional file

Additional file 1: SPIRIT checklist for the RePST trial, indicating which manuscript page contains each element of the study protocol. (DOX 135 kb)

## Abbreviations

CAA: Circulating anodic antigen
CCA: Circulating cathodic antigen
CI: Confidence interval
CR: Cure rate
CRF: Case report form
CSRS: Centre Suisse de Recherches Scientifiques
Ct: Cycle threshold value
DNA: Deoxyribonucleic acid
DSMB: Data safety and monitoring board
EPG: Eggs per gram of faeces
HDSS: Health and demographic surveillance system
IRR: Intensity reduction rate
KK: Kato-Katz technique
LCA: Latent class analysis
LF: Lateral flow
LUMC: Leiden University Medical Center
MDA: Mass drug administration
NPV: Negative predictive value
PCR: Polymerase chain reaction
PNLMTN-CP: Programme National de Lutte contre les Maladies Tropicales Négligées à Chimiothérapie Préventive
POC: Point-of-care
PPV: Positive predictive value
PZQ: Praziquantel
RePST: Repeated doses of praziquantel in schistosomiasis treatment in Schiss
UCP: Up-converting phosphor particles
UF: Urine-filtration technique
WHO: World Health Organization
